# Primary Peritoneal Cancer Two Decades after a Bilateral Salpingo-Oophorectomy

**DOI:** 10.1155/2019/1870834

**Published:** 2019-03-25

**Authors:** Melinda Rodowa, Amir H. Salehi, Jacob McGee

**Affiliations:** ^1^Department of Obstetrics & Gynecology, Western University, London, Canada; ^2^Pathology and Molecular Medicine, Juravinski Hospital, Hamilton, Canada

## Abstract

Bilateral salpingo-oophorectomy (BSO) is increasingly employed as a risk-reducing strategy for epithelial ovarian cancer (EOC). We report the third case of a patient developing primary peritoneal cancer two decades after a bilateral salpingo-oophorectomy. This 66-year-old female underwent a hysterectomy for pelvic pain at age 28 and a subsequent bilateral salpingo-oophorectomy (BSO) at age of 45 for a pelvic mass. Presenting with a 6-month history of increasing abdominal girth, decreased energy, and a reduction in appetite, she was consented for a unilateral salpingo-oophorectomy, omentectomy, and cytoreductive surgery. Pathology specimens revealed a high grade metastatic papillary serous carcinoma consistent with a primary gynecologic origin. It is unlikely that an occult malignancy was missed at the time of pathologic assessment following her previous BSO; therefore it provides evidence that primary peritoneal cancers can arise through the malignant transformation of benign endosalpingiosis.

## 1. Introduction

In North America, ovarian cancer has the highest case fatality rate amongst gynecological malignancies. In Canada each year, 1750 women will die from ovarian cancer [1]. The lifetime risk of developing epithelial ovarian cancer (EOC) is 1.8%, with a significantly higher risk for BRCA mutation carriers ranging from 15 to 54% [2, 3].

A risk-reducing strategy employed for women with a strong family history of ovarian carcinoma/BRCA mutation carriers is prophylactic bilateral salpingo-oophorectomy (BSO). In addition, BSO at the time of hysterectomy for benign conditions has been suggested to reduce the risk of EOC, particularly in postmenopausal women [4]. Furthermore, some have advocated for bilateral salpingectomy at the time of surgery for benign conditions as a risk-reducing strategy [5]. In high risk patients, including women with BRCA1/2 and HNPCC mutations, the risk of primary peritoneal carcinoma remains after prophylactic BSO.

## 2. Case Description

A 66-year-old female presented to the gynecology oncology clinic with a 6-month history of increasing abdominal girth, decreased energy, and a reduction in appetite. She reported a twenty pound weight gain over the preceding two months.

The patient reported a previous hysterectomy at age 28 for pelvic pain and then a bilateral salpingo-oophorectomy at age 45 for a pelvic mass. Past medical history was significant for atrial fibrillation, type 2 diabetes mellitus, hypertension, and gastro-esophageal reflux disease. Her family history was significant for a maternal aunt with a diagnosis of breast cancer at age 58 and subsequent ovarian cancer at age 90.

A diagnostic paracentesis was performed preoperatively and returned positive for an adenocarcinoma. An abdominal CT scan revealed a query 4.8 x 2.1 cm left ovarian cyst and omental cake. CA-125 was elevated at 278 U/mL (Normal High <=35). On examination, BMI was 38. Auscultation of the heart and lungs was normal. Abdominal examination showed shifting dullness. Rectovaginal examination revealed ascites, but no pelvic mass or nodularity in the cul-de-sac.

With a presumed diagnosis of ovarian cancer, the patient was presented with two options, neoadjuvant chemotherapy, or primary cytoreductive surgery. After a discussion of risks and benefits of each approach, the patient consented for primary cytoreduction.

A laparotomy was performed, and upon entering the peritoneal cavity, diffuse inflammation of the peritoneum and 12L of ascites were noted. Extensive carcinomatosis involving most peritoneal surfaces was identified with involvement of the ascending colon. Preoperatively we had queried the possibility of some residual ovary; however no ovary could be identified within the peritoneal cavity, nor with dissection into the retroperitoneum. A supracolic omentectomy, peritoneal stripping of both upper abdomen and pelvis, and right sided hemicolectomy with primary reanastomosis were performed.

In the postoperative period, the patient developed rapid atrial fibrillation and became persistently hypotensive despite aggressive fluid resuscitation. She was brought to the intensive care unit for resuscitation, but continued to decline. At the patient's request, treatment was withdrawn; she died shortly thereafter.

Pathology specimens revealed a high grade metastatic papillary serous carcinoma consistent with a primary gynecologic origin. The patient's previous surgical and pathology reports were obtained. The operative note from 1993 described that the patient had undergone a laparotomy with removal of both fallopian tubes and ovaries. Final pathology from that surgery confirmed removal of a normal left ovary and tube and the removal of a right ovary (containing a serous cyst) and a normal right fallopian tube. No borderline or malignant changes were identified.

## 3. Discussion

To our knowledge this is only the third case of a patient developing primary peritoneal cancer two decades after a BSO. A previous report by Casey et al. described a 55-year BRCA1 mutation carrier who developed primary peritoneal cancer 24.3 years following prophylactic salpingo-oophorectomy. Investigation of this patient's ovaries revealed no malignant or borderline changes [6]. Piver described a similar case of a 75-year-old patient with unknown mutation status, but a family history of ovarian cancer in four first degree relatives, who developed primary peritoneal cancer 27 years following BSO. This patient's ovaries were also reported as normal at the time of removal [2].

For women with a BRCA mutation, the literature quotes a consistent risk of 3-4% risk of primary peritoneal cancer following prophylactic salpingo-oophorectomy over twenty years, with the vast majority of cases occurring within 3-4 years [3, 6–8]. The risk appears to be higher for known BRCA1 mutation carriers as opposed to BRCA2 mutation carriers (3.9 versus 3.5) [6].

Of the two prior cases presenting with primary peritoneal cancer two decades after prophylactic BSO, one patient was a known BRCA1 mutation carrier and the other patient had a significant family history for ovarian cancer. Unfortunately, the mutation status of our patient was unknown, as the patient passed away before any further treatment or genetic testing could be offered. The patient is a possible mutation carrier as there was a known family history of ovarian and breast cancer in a second degree relative. Furthermore, a somatic BRCA mutation at the tumoral level is a possibility.

Regarding the pathogenesis of high grade serous tumors (HGST) of pelvic origin, a model gaining wide acceptance proposes that these tumors originate in fallopian tube epithelium (FTE) as premalignant growths dubbed STICs (serous tubal intraepithelial carcinomas) which undergo malignant transformation and, due to their location, extend to nearby ovary and surrounding pelvic peritoneum [9, 10]. Supporting this hypothesis are the large number of studies demonstrating presence of occult noninvasive (i.e., STICs) and invasive carcinomas of the fallopian tube, in about half of patients with concurrent HGST of the ovary or in those undergoing prophylactic BSO due to BRCA mutations status. Similarly, in cases of primary peritoneal carcinomas, nearly half have been found to be associated with a STIC, when the fallopian tube is well examined [11].

Conversely, in about half the cases of pelvic HGST, there are no concurrent fallopian tube neoplastic changes. This has led to the proposal that both low and high grade serous carcinomas can arise from benign fallopian tube epithelial (FTE) inclusions in the ovary (cortical inclusion cysts) or on peritoneal surfaces (endosalpingiosis) which undergo malignant transformation independently of changes in the fallopian tube [12]. These initially benign FTE inclusions, in turn, have been shown to arise from the sloughing of normal FTE during ovulation [13].

In our case, the interval between BSO and the later development of primary peritoneal cancer was 21 years, making it unlikely that an occult malignancy was missed at the time of pathologic assessment following her BSO at the age of 45. We reviewed the pathology report from her original surgery, and it confirmed removal of both ovaries and fallopian tubes. Assuming no residual ovary/fallopian tube remained, the malignancy likely arose from benign peritoneal FTE inclusions (endosalpingiosis) that had been sloughed from the patient's fallopian tubes before these were removed, subsequently undergoing malignant transformation many years later. It therefore provides evidence that primary peritoneal cancers can arise through the second mechanism described above, through the malignant transformation of benign endosalpingiosis. It further demonstrates that fallopian tube STICs are not a necessary intermediary step in the pathogenesis of primary peritoneal carcinomas.

## 4. Conclusion

This case demonstrates a rare presentation of primary peritoneal cancer presenting twenty-one years after bilateral salpingo-oophorectomy.
